# Microbial and physicochemical water quality changes within distribution and premise plumbing systems during a chlorine conversion

**DOI:** 10.1371/journal.pwat.0000181

**Published:** 2024-02-08

**Authors:** Helen Y. Buse, Jatin H. Mistry

**Affiliations:** 1 Office of Research and Development, United States Environmental Protection Agency, Cincinnati, Ohio, United States of America; 2 Drinking Water Section, United States Environmental Protection Agency Region 6, Dallas, Texas, United States of America

## Abstract

A strategy for nitrification control within chloraminated drinking water systems (CDWSs) is to temporarily switch from chloramine secondary disinfection to free chlorine, also known as a free chlorine conversion (FCC). However, the long-term and beneficial effects of FCCs are unclear, especially regarding opportunistic pathogen occurrence. In this study, the impacts to microbial and physicochemical parameters were monitored throughout a CDWS implementing a FCC. Water samples were collected weekly for 4–6 weeks before, during, and after a FCC at eight locations: four distribution system and four residential sites. Monochloramine residual (mean±standard deviation) before and after the FCC averaged 1.8±0.9 and 1.6±1.0 parts per million (ppm) for all sites, respectively. Free chlorine levels averaged 2.3±0.9 ppm. There were no significant differences in turbidity and hardness at each location during the three time periods, but some were noted for pH, temperature, and orthophosphate levels across various sites and sampling periods. For all locations, heterotrophic plate count levels were lower during the FCC compared to the periods before and after. All samples from one residence were culture positive for *P*. *aeruginosa* which exhibited high levels before the FCC, decreasing levels during, and steadily increasing levels after. Additionally, one week prior to the FCC, sediment samples from two elevated storage tanks, ET-1 and ET-2, were analyzed with ET-1 displaying higher levels of culturable heterotrophic bacteria and molecularly detected total bacteria, *Legionella* spp., and nontuberculous mycobacteria (NTM), as well as presence of culturable *P*. *aeruginosa* and total coliforms compared to ET-2. Fourteen *P*. *aeruginosa* and total coliform isolates were whole genome sequenced with genetic differences observed depending on the sampling location and timepoint. Collectively, the observed differences in chemical and microbial parameters advocates for a better understanding of the effects associated with implementing FCCs to determine both their effectiveness and potential risks/rewards to water quality.

## 1. Introduction

Chlorine, chloramines, chlorine dioxide, and ozone are commonly used as oxidizing disinfectants for microbiological control in drinking water, whose importance, applications, and efficacy assessments have been studied and discussed extensively [1, 2]. Primary disinfection occurs within the drinking water treatment process while secondary disinfection is applied to maintain a disinfectant residual, control microbial regrowth, and reduce risks from microbial pathogens throughout the drinking water distribution system. The advantages of using chloramines as a secondary disinfectant include stability and maintenance of disinfectant residual [3], lower total trihalomethane (TTHM) and haloacetic acid (HAA) disinfection byproduct (DBP) formation potential [4], greater biofilm penetration [5], and reduction in the occurrence of *Legionella* within premise plumbing systems [6]. However, more recent studies have reported that the greater biofilm penetration of chloramines did not correlate with loss of microbial viability and decreased biofilm material [7]. Moreover, the advantages of chloramines may be offset because they have been shown to: decay as quickly as chlorine [8], result in increased formation of iodinated- and nitrogen-containing DBPs [9], inactivate biofilm-associated *L*. *pneumophila* differently depending on the underlying pipe material type [10], and select for chloramine tolerant *Mycobacterium* species [11].

A recent 2017 survey of 375 drinking water systems in the United States indicated that 65% of systems utilized chlorine as a secondary disinfectant and 25% used chloramines, with the latter showing a decrease from 30% in the previous 2007 survey of 312 systems [12, 13]. These chloraminated drinking water system (CDWS) survey respondents indicated a concern about balancing DBPs and simultaneous compliance, public perception issues regarding chloramine usage, and nitrification [13].

In CDWSs, nitrification is indeed a major concern and is caused by microbially mediated oxidation of ammonia to nitrite, and then nitrite to nitrate. Nitrification can also occur with complete oxidation of ammonia to nitrate [14]. Chemical, microbial, and aesthetic water quality is negatively impacted by nitrification as it leads to loss of disinfectant residual; dissolved oxygen depletion; reduction in pH and alkalinity; DBP formation due to implementation of control strategies; production of nitrite and/or nitrate; increases in heterotrophic growth, coliform occurrences, and nitrifying microorganisms; along with taste and odor, color, and turbidity concerns [15]. Within premise plumbing systems, degradation of water quality due to nitrification can be more pronounced than that observed in the overall distribution system because of intermittent water usage, longer water stagnation times, elevated temperatures, and presence of copper and polyvinyl chloride (PVC) piping materials [16, 17].

CDWSs typically have a nitrification monitoring program or a nitrification control/action plan in place. Water quality parameters, such as pH, temperature, free chlorine, monochloramine, total or combined chlorine, free ammonia, nitrite/nitrate, and heterotrophic plate counts (HPC) can be used for nitrification monitoring and detection [13, 15]. Nitrification control can include operational (e.g., manual or automated flushing to minimize water age and maintaining disinfectant residual in the distribution system) and treatment practices (e.g., removing, or controlling for, excess ammonia). CDWSs typically undergo a free chlorine conversion (FCC), or chlorine burn, which is a temporary secondary disinfectant change from chloramines to free chlorine resulting in the oxidation of ammonia and thus, removal of this substrate for nitrifying bacteria. It is not unusual for a water system to conduct their FCC outside of their regular DBP compliance monitoring period since formation and elevated levels of DBPs are expected to occur during the FCC [18]. The frequency and duration of FCCs can vary by utility. Most drinking water systems in EPA R6 conduct a FCC once a year, can be initiated as early as Winter to Fall, and can last between 30–60 days. However, most drinking water systems do not conduct a FCC more than once a year due to the extensive preparation required, public notifications, and consumer complaints (e.g., inconveniences to homeowners with fish tanks, dialysis centers, and other customers, such as industries, that demand a certain water quality).

Despite the frequent use of FCCs, the effectiveness and benefits of this practice along with changes to drinking water chemistry, microbiology, and overall water aesthetics, before, during, and after an FCC are not well studied, especially within both the distribution and premise plumbing systems. In this study, microbial and physicochemical water quality parameters were monitored throughout a surface water CDWS implementing a FCC to understand the impacts and assess changes in water quality.

## 2. Materials and methods

### 2.1 Description of the drinking water treatment plant and processes

Samples used in this study were collected throughout a CDWS supplied by a surface water treatment plant utilizing conventional treatment processes (coagulation, sedimentation, filtration, and disinfection). No permits were required for this work as authorized water treatment plant personnel conducted the field sampling and performed on-site water quality measurements. For corrosion control and sequestration of iron and manganese in the system, the treatment plant utilizes an approximately 1:3 orthophosphate to polyphosphate blend, applied at a 1.25 parts per million (ppm) concentration, after filtration. In this study, the average ± standard deviation (SD) level of orthophosphate leaving the treatment plant was 0.27 ± 0.03 ppm, which was within the utility’s targeted range of 0.25–0.28 ppm. While chlorine is the primary plant disinfectant, chloramine is added prior to water entering the distribution system. This system undergoes a free chlorine conversion (FCC) for approximately 30 to 60 days every year. During this study, the FCC lasted 42 days.

### 2.2 Description of sampling locations

**Fig 1.**
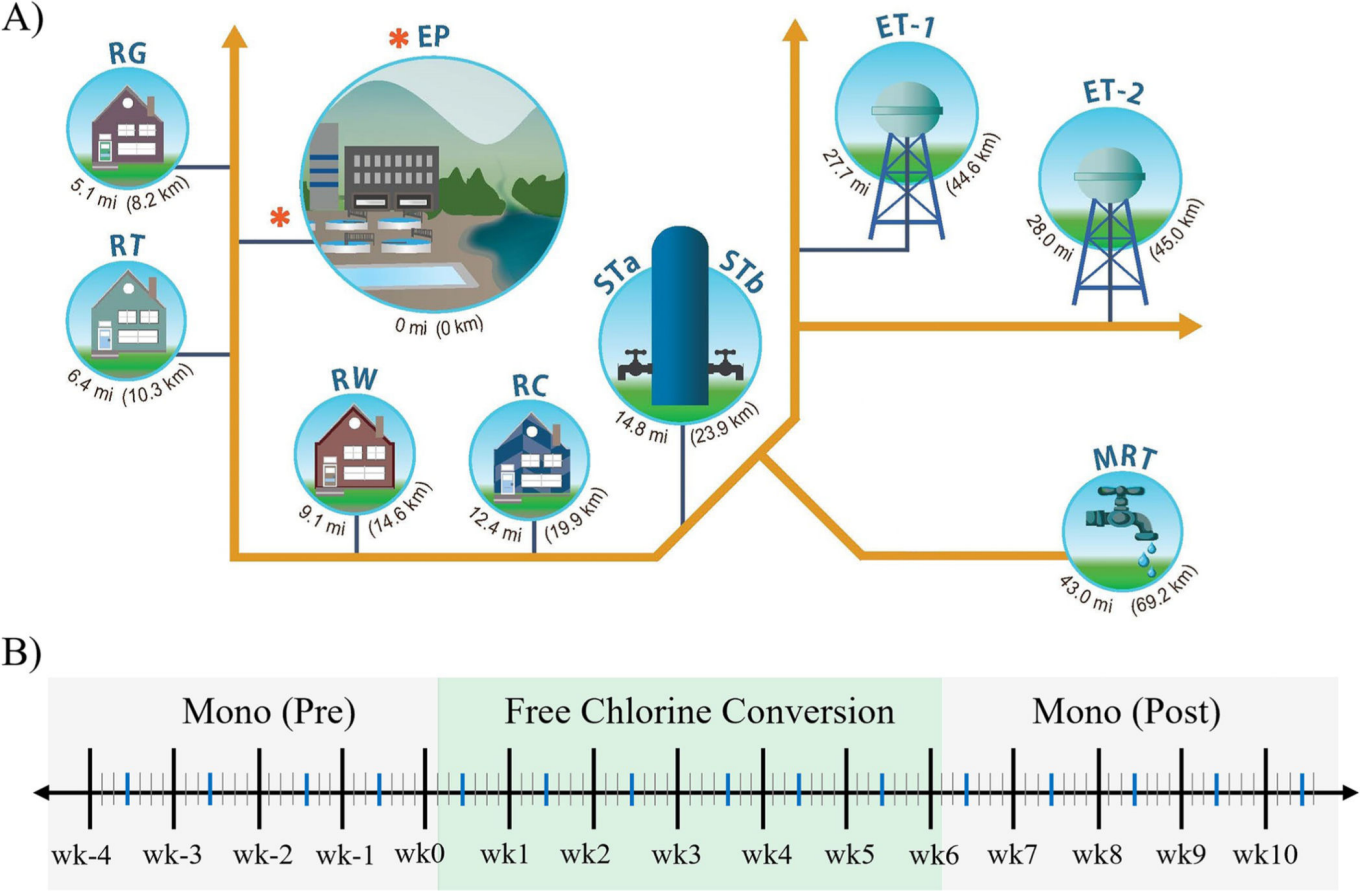
Drinking water distribution system sampling locations and timeline. (A) Distribution system sites: finished water entry point (EP), storage tank inlet (STa) and outlet (STb); and elevated storage tanks, ET-1 and ET-2. Residential (R), premise plumbing sampling sites: RG, RT, RW, and RC. Relative distance of each site from the EP, below each graphic. (B) Sampling timeline: weeks (black vertical lines), days (short grey vertical lines), microbial sampling days (vertical blue lines). Mono(Pre), chloramine disinfectant residual period prior to the Free Chlorine Conversion (FCC). FCC, free chlorine disinfectant residual period. Mono(Post), period after the FCC and return to chloramine disinfectant residual.

Water samples were collected at eight locations: the entry point (EP) of treated water into the distribution system, from a storage tank inlet (STa) and outlet (STb), at a maximum hydraulic residence time (MRT) location, and four premise plumbing residential sites, designated RC, RG, RT, and RW (Fig 1A). EP and MRT are regulatory compliance monitoring locations for the CDWS used in this study. Locations STa and STb were chosen to evaluate the impacts of a storage tank on water quality. Residential locations were incorporated in this study to evaluate water quality at the point of use and thus, would have more public health implications. Sediment samples from ET-1 and ET-2 were only collected once (one week prior to the FCC) when the storage tanks were drained and cleaned in preparation for the FCC. EP, STa and STb, and MRT are established compliance monitoring site locations in the distribution system. Fig 1B illustrates the sampling timeline periods. Prior to the FCC, when chloramine was the disinfectant residual, abbreviated here as Mono (Pre), samples were collected weekly for four weeks (wk-4 to wk-1). During the FCC period, samples were collected weekly for 5 weeks (wk0 to wk5). When the system converted back to using a chloramine residual, abbreviated here as Mono (Post), samples were collected weekly for another 5 weeks (wk6 to wk10). In this study, the kitchen faucet served as the sampling outlet for the premise plumbing residential sites (S1 Fig).

### 2.3 Sample collection and processing

#### 2.3.1 Bulk water.

At each distribution system sampling location (EP, STa, STb, and MRT), bulk water samples were collected after a 1–3 min flush. For residential locations (RG, RC, RT, and RW), cold bulk water samples were collected from the kitchen after an overnight stagnation period of at least 6 h. 100 mL were collected for immediate onsite water quality analysis described in Section 2.4, followed by 2 L from the EP, STa, STb, and MRT locations and from the kitchen faucet at the residential sites, RG, RC, RT, and RW. Cold bulk water samples were collected in sterile 1L plastic bottles containing 1 mL of 10% w/v sodium thiosulfate to neutralize any disinfectant residual. Sample bottles were shipped overnight on wet ice (≤ 4°C) and were processed within less than 24 h after collection.

1 L of each sample was filtered through a 0.2 μm polyethersulfone membrane (Supor Membrane, Pall Life Sciences, Nassau, NY, USA). Filters were placed into 10 mL of dechlorinated, 0.22 μm filtered drinking water (dfH_2_O), and vortexed at maximum speed for 1 min to resuspend the concentrated bulk water material. Approximately 1 mL of the concentrated bulk water suspension was analyzed for *Legionella* spp. colony forming unit (CFU), as described in Section 2.5.2. For wk1 samples, collected during the FCC period, 750 mL of the bulk water samples were filtered through a 0.4 μm polycarbonate membrane (Pall Life Sciences). Membranes were placed in Lysing Matrix A tubes (MP Biomedicals, Solon, OH, USA) for nucleic acid extraction as described in Section 2.6.

#### 2.3.2 Storage tank sediment.

One week prior to the FCC period, two elevated storage tanks in the distribution system were emptied and cleaned, ET-1 and ET-2 (Fig 1A). Storage tank sediment samples were collected into four sterile 1L bottles from ET-1 and two 1L bottles from ET-2 (S2 Fig). The liquid phase from each bottle, which contained particulate sediment matter (S2E and S2F Fig), was decanted into separate sterile containers, and processed for microbial analyses as described in Section 2.5. Five hundred microliters of the sediment samples were placed in a Lysing Matrix A tube (MP Biomedicals, Solon, OH, USA) for nucleic acid extraction as described in Section 2.6. Mass of each aliquot was recorded to express results as units per gram (g-^1^).

### 2.4 Water quality analysis

Bulk water samples were analyzed for physicochemical parameters (pH, temperature, turbidity) and chemical parameters (monochloramine, free and total chlorine, free ammonia, orthophosphate, nitrite, and hardness). pH, temperature, monochloramine, free and total chlorine, free ammonia, orthophosphate, and nitrite were analyzed onsite using the Hach SL1000 Portable Parallel Analyzer and Chemkey reagents following manufacturer’s instructions (Hach, Loveland, CO, USA). After laboratory receipt of the shipped samples, turbidity and hardness were measured using a Hach 2100Q Portable Turbidimeter and EDTA titration kit, respectively (Hach, Loveland, CO, USA).

### 2.5 Microbial culture analysis

#### 2.5.1 Heterotrophic plate count (HPC).

HPCs in unconcentrated bulk water and storage tank sediment samples were enumerated using the spread plate method on Reasoner’s 2A (R2A) and Plate Count (PC) agar (Difco Laboratories, Detroit, MI, USA). R2A plates were incubated at 28°C for 7 d and PC plates at 35°C for 48 h [19].

#### 2.5.2 *Legionella* spp..

*Legionella* enumeration and presumptive colony analysis was performed as previously described [20] and following ISO 11731 [21]. Briefly, undiluted and serially diluted suspensions, of bulk water and sediment samples, were spread plated on buffered charcoal yeast extract (BCYE) agar plates (BD Diagnostics, Franklin Lakes, NJ, USA) and incubated for 4–6 days at 36°C. A portion of the sample was also heat treated, by incubating in a 50°C water bath for 30 min, before plating on BCYE agar plates. A 100 mL portion of unconcentrated bulk water samples collected from RG, RT, RW, and RC at wk4 (Fig 1) was analyzed using Legiolert (IDEXX, Westbrook, ME, USA) following manufacturer’s instructions. One milliliter of the storage tank sediment sample was added to 99 mL of Butterfield’s phosphate buffer (Hardy Diagnostics, Santa Maria, CA, USA) and analyzed using Legiolert, not in accordance with manufacturer’s protocols since tank sediment is neither a potable nor non-potable water sample. Presumptive *Legionella* colonies and Legiolert positive wells were isolated and confirmed as *Legionella* spp. or *L*. *pneumophila* via polymerase chain reaction (PCR) using the 16S rRNA gene assays described in Section 2.7.

#### 2.5.3 *Pseudomonas aeruginosa*, Total Coliform (TC), and *Escherichia coli*.

Pseudalert and Colilert (IDEXX) were used to analyze 100 mL of the unconcentrated bulk water samples according to the manufacturer’s instructions. One milliliter of the storage tank sediment samples was added to 99 mL of Butterfield’s phosphate buffer and analyzed, which was not in accordance with manufacturer’s protocols. Presumptive positive wells from the Pseudalert trays were extracted and confirmed as *P*. *aeruginosa* via polymerase chain reaction (PCR) using the *ecfX* gene assay described in Section 2.7. Well extracts from the Pseudalert and Colilert positive wells were streaked onto a Tryptic Soy Agar (TSA; BD Company) plates and incubated for 1–3 days at 36°C. TSA plates were checked for purity and a single colony was used to inoculate 10 mL of Tryptic Soy Broth (TSB; BD Company) and incubated for 15–18 h with shaking at 36°C. One milliliter of the stationary phase culture was (i) pelleted and processed for total DNA extraction as described in Section 2.6 and (ii) washed, resuspended in 1mL TSB with 10% glycerol, and stored at −80°C.

#### 2.5.4 Limit of detection (LOD).

To account for zero values, 1 was added to all data points before conversion to the log_10_ scale (e.g., log_10_ (CFU + 1)). For HPCs, the LOD for bulk water samples was 10, or 1 log_10_, CFU mL^−1^. For *Legionella* spp., the LOD for bulk water samples was 1, or 0 log_10_, CFU Ml^−1^, and for sediment samples 10, or 1 log_10_, CFU g^−1^. For Legiolert, the LOD was 10 MPN 100mL^−1^ with a quantification limit of 22,726 MPN 100mL^−1^ for bulk water samples. The LOD for the Quanti-Tray/2000, used for Pseudalert and Colilert, was 1 MPN 100mL^−1^ with a quantification limit of 2,419.6 MPN 100mL^−1^. For sediment samples, the LOD and quantification limit for Legiolert was 100 and 227,260 MPN g^−1^, respectively; and for Pseudalert and Colilert, LOD was 100, or 2 log_10_, MPN g^−1^ and the quantification limit 241,960, or 5.4 log_10_, MPN g^−1^.

### 2.6 Isolation and preparation of total DNA

DNA was extracted using the MasterPure Complete DNA purification kit (Epicentre Biotechnologies Inc., Madison, WI, USA) according to manufacturer’s protocol. Samples were homogenized using the FastPrep-24 bead beating and lysing system (MP Biomedicals, Solon, OH, USA) and processed twice for 30 s and oscillated at a speed of 4 meters s^−1^. The DNA pellet was resuspended in 100 μL of molecular grade water (Corning, Manassas, VA, USA).

### 2.7 Quantitative polymerase chain reaction (qPCR)

*Legionella* spp., *L*. *pneumophila*, and *Vermamoeba vermiformis* qPCR was performed as previously described [20]. TaqMan qPCR assays for detection of total bacteria targeted the 16S rRNA gene [22]; for *P*. *aeruginosa*, the extracytoplasmic function sigma factor, *ecfX*, gene [23]; for nontuberculous mycobacteria (NTM), the heat shock protein 65, *hsp65*, gene [24]; and for *Acanthamoeba* spp. and *Naegleria fowleri*, their respective, 18S rRNA gene [25].

DNA samples were analyzed in duplicate using the Applied Biosystems QuantStudio 6 Flex Fast Real-Time PCR system (ThermoFisher, Waltham, MA, USA). A 10-fold dilution of each sample was also analyzed in duplicate to test for presence of environmental qPCR inhibitors. For all microbial targets, standard curves were generated, on each plate, using a plasmid vector (pUCIDT-AMP; Integrated DNA Technologies, Inc., Coralville, IA, USA) containing a cloned region of the gene target. Standards ranging from 1 to 10^7^ gene copies (GC) for each target were generated and analyzed in triplicate along with duplicate no-template controls for each 96-well plate. The limits of detection for bulk water and storage tank sediment supernatant samples were 1.3 log_10_ GC mL^−1^ and 1.3 log_10_ GC g^−1^, respectively.

### 2.8 Whole genome sequencing and analysis

Fourteen presumptive *P*. *aeruginosa*, total coliform, and *E*. *coli* isolates were chosen for whole genome sequencing. Total genomic DNA from each isolate was prepared as described above. DNA extracts were quantified using an Invitrogen Qubit 4 Fluorometer and 1x dsDNA High Sensitivity Assay Kit (ThermoFisher Scientific, Waltham, MA, USA). Metagenomic libraries were prepared using the DNA extracts and the Nextera XT DNA Library Preparation kit (Illumina, San Diego, CA, USA). Libraries were quality checked with an Agilent 2100 Bioanalyzer and DNA High Sensitivity kit and pooled in an equimolar ratio. The pool was gel purified using a 2% agarose gel and the Qiagen QIAquick gel extraction kit (Qiagen, Germantown, MD, USA). Following purification, the pool was sequenced by Wright Labs (Huntingdon, PA, USA) using an Illumina NextSeq 550 to produce 2×150 bp reads. The Illumina reads are deposited in the National Center for Biotechnology Information (NCBI) Sequence Read Archive (SRA) database under the BioProject accession number PRJNA871216 (Temporary Submission ID: SUB11952375 Release date: 2023–10-31).

Prior to assembly, raw sequences were quality checked with FastQC v0.11.9 (Babraham Institute, UK, http://www.bioinformatics.babraham.ac.uk/projects/fastqc/). Libraries were (i) cleaned from contaminants, adapters, and other Illumina-specific sequences from the reads; (ii) removed of low coverage reads; (iii) filtered to a minimum length read of 100 nt; and (iv) assembled using Trimmomatic v0.36 [26], PRINSEQ v0.20.4 [27], and SPAdes v3.15.3 [28]. Taxonomic identification on assembled genomic sequences were assigned using GTDB-Tk v1.7.0 [29] and verified with the average nucleotide identity (ANI) tool [30]. Assembled genomes were annotated with Prokka v1.14.5 [31] and quality checked with CheckM v1.0.18 [32]. Phylogenetic trees were constructed using FastTree2 [33]. Genomes were also annotated with rapid annotations using subsystems technology (RAST) and the comparative genomics environment, SEED, was used to examine the general functional gene distribution, specific functional gene families, and protein domains within genome sets [34, 35].

### 2.9 Statistical analysis

The Shapiro–Wilk normality test was conducted on the datasets collected during the Mono (Pre), FCC, and Mono (Post) sampling periods for each analyte at each sampling location to determine distribution of the data throughout that sampling period. A one-way analysis of variance (ANOVA) using the Tukey multiple comparisons test was conducted between analytes, with *P*-values of < 0.05 considered statistically significant. Two-tailed paired t-tests (parametric) were used to compare HPC levels using the R2A and PC method. All statistical analyses were performed using Prism v8.4.3 (GraphPad Software, San Diego, CA, USA).

## 3. Results

### 3.1 Physiochemical water quality during the study period

Two 1L water samples were collected weekly from eight locations throughout a chloraminated drinking water system (CDWS) (Fig 1A). Sampling occurred for 15 weeks and spanned three distinct time periods: before, during, and after a 42-day free chlorine conversion (FCC) (Fig 1B). S3 Fig shows the pH, temperature, turbidity, and hardness levels observed throughout the study. Average±SD pH levels at the RT site before, during, and after the FCC were 7.4±0.1, 6.9± 0.3, and 6.7±0.04, respectively, with levels before the FCC statistically higher than the levels during and after the FCC (*P* < 0.05). For all locations, there were no statistically significant differences in their respective pH, turbidity, and hardness levels across the three sampling periods, except for pH at the RT site (S3D Fig).

Within the distribution system, temperatures were statistically lower before the FCC compared to after: 28±2 v 32±2°C at EP (*P* = 0.0004), 26±1 v 30±0.1°C at STa (*P* = 0.001), 26±1 v 29±1°C at STb (*P* = 0.0017), and 25±1 v 28±0.5°C at MRT (*P* = 0.0054). The different temperatures observed before and after the FCC at these sites most likely reflected the progression of sampling into the warmer summer months. At the residential locations, statistically different temperatures were observed at the RT site before and during the FCC (25±1 and 28±1°C, *P* = 0.0185), at the RW site before and after the FCC (24±1 and 28±1°C, *P* = 0.0012), and at the RG site both before and after the FCC (26±1 v 23±0.4°C, *P* < 0.05) as well as during and after the FCC (27±2 v 23±0.4°C, *P* < 0.0001). Temperature differences at the residential locations most likely reflected a combination of seasonal effects and variations in indoor ambient conditions (S3B and S3E Fig).

**Fig 2.**
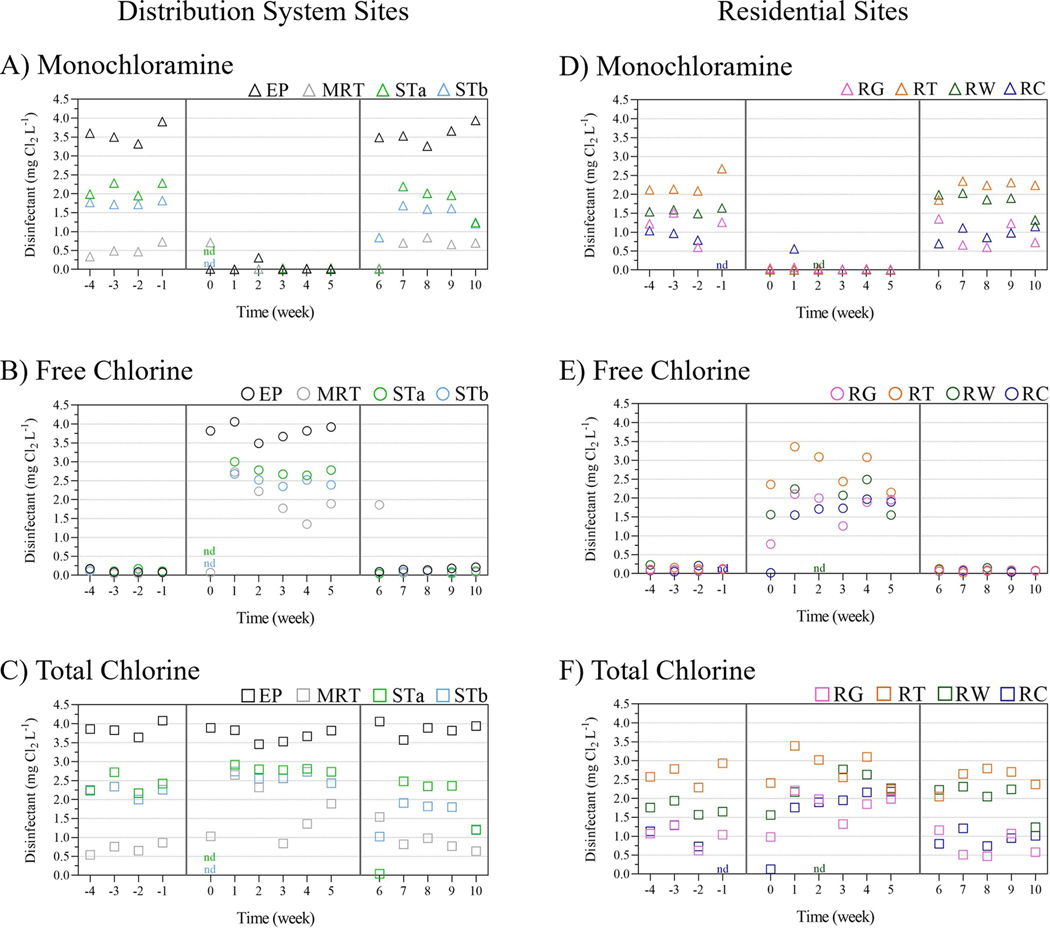
Temporal disinfectant residual summary for distribution (A-C) and residential (D-F) sites. Sampling occurred weekly before (wk-4 to wk-1), during (wk0 to wk5), and after (wk6 to wk10) the FCC period. Monochloramine (A, D), free chlorine (B, E), and total chlorine (C, F) levels are shown in triangle, circle, and square symbols, respectively. Each sampling location is represented by different colors (EP, black; MRT, grey; STa, light green; STb, light blue; RG, pink; RT, orange; RW, dark green; RC, dark blue). nd, no data, for STa and STb during week 5, for RC during week 4, and for RW during week 7.

The monochloramine, free chlorine, and total chlorine residual levels observed throughout the study are shown in Fig 2.

At each location, their respective monochloramine and total chlorine levels, before (wk-4 to wk-1), during (wk0 to wk5), and after (wk6 to wk10) the FCC, were not statistically different (*P* > 0.11). The average ± standard deviation (SD) in parts per million (ppm) of monochloramine residual was 3.6±0.2 at the EP location, 1.8±0.7 at STa, 1.6±0.3 at STb, and 0.5±0.2 at MRT for the distribution system locations (Fig 2A) and 1.0±0.4 at RG, 2.2±0.2 at RT, 1.7±0.2 at RW, and 1.0±0.2 at RC for the residential locations (Fig 2D). Due to the hydraulic distance of the MRT location from the treatment plant, the monochloramine residual at MRT was 0.71 ppm at wk0, 48 hr after the conversion to free chlorine (Fig 2A, grey circle at wk0). Similarly, at wk6, after the conversion back to monochloramine, the free chlorine residual at MRT was 1.86 ppm, (Fig 2B, grey circle at wk6).

Free chlorine levels for all sites before and after the FCC were 0.1±0.04 and 0.1±0.3 (Fig 2B and 2E). During the FCC, free chlorine levels (average ± SD ppm) at the EP site (3.8±0.2) were statistically higher than those at the STa (2.8±0.1), STb (2.5±0.1), MRT (1.7±0.9), RG (1.7 ±0.5), RT (2.7±0.5), RW (2.0±0.4), and RC (1.5±0.1) locations (*P* < 0.0001, Fig 1B and 1E). The RT site had the highest residential free chlorine level and was statistically higher than those at the RG and RC locations (*P* < 0.05).

Free ammonia, nitrite, and orthophosphate levels were also monitored throughout the study (S4 Fig). Free ammonia levels at the RG site were higher than levels during the FCC (0.38±0.04 v 0.10±0.09 ppm, *P* = 0.0047). Nitrite levels at the MRT site, before the FCC (70.6 ±14.7 ppb), were higher than levels during (16.0±17.3 ppb) and after (7.4±0.5 ppb) the FCC (*P* < 0.0001). For all locations, there were no statistically significant differences in their respective nitrite and free ammonia levels across the three sampling periods, except for nitrite levels at MRT (S4B Fig) and free ammonia levels at RG (S4D Fig).

Orthophosphate levels were statistically the lowest at EP (0.27±0.03 ppm, *P* < 0.001) and highest at MRT (0.61±0.08 ppm, *P* < 0.0001) amongst the distribution system locations. For the residential sites, orthophosphate levels at RT (0.38±0.07 ppm, *P* < 0.05) and RG (0.64 ±0.08 ppm, *P* < 0.01) were statistically the lowest and highest, respectively (S4C and S4F Fig) even though RG is closer to the EP location compared to the other residential sites. Moreover, there were no statistical differences between the orthophosphate levels at the MRT and RG locations (*P* = 0.7350).

### 3.2 Analysis of sediment samples from elevated storage tanks

**Fig 3.**
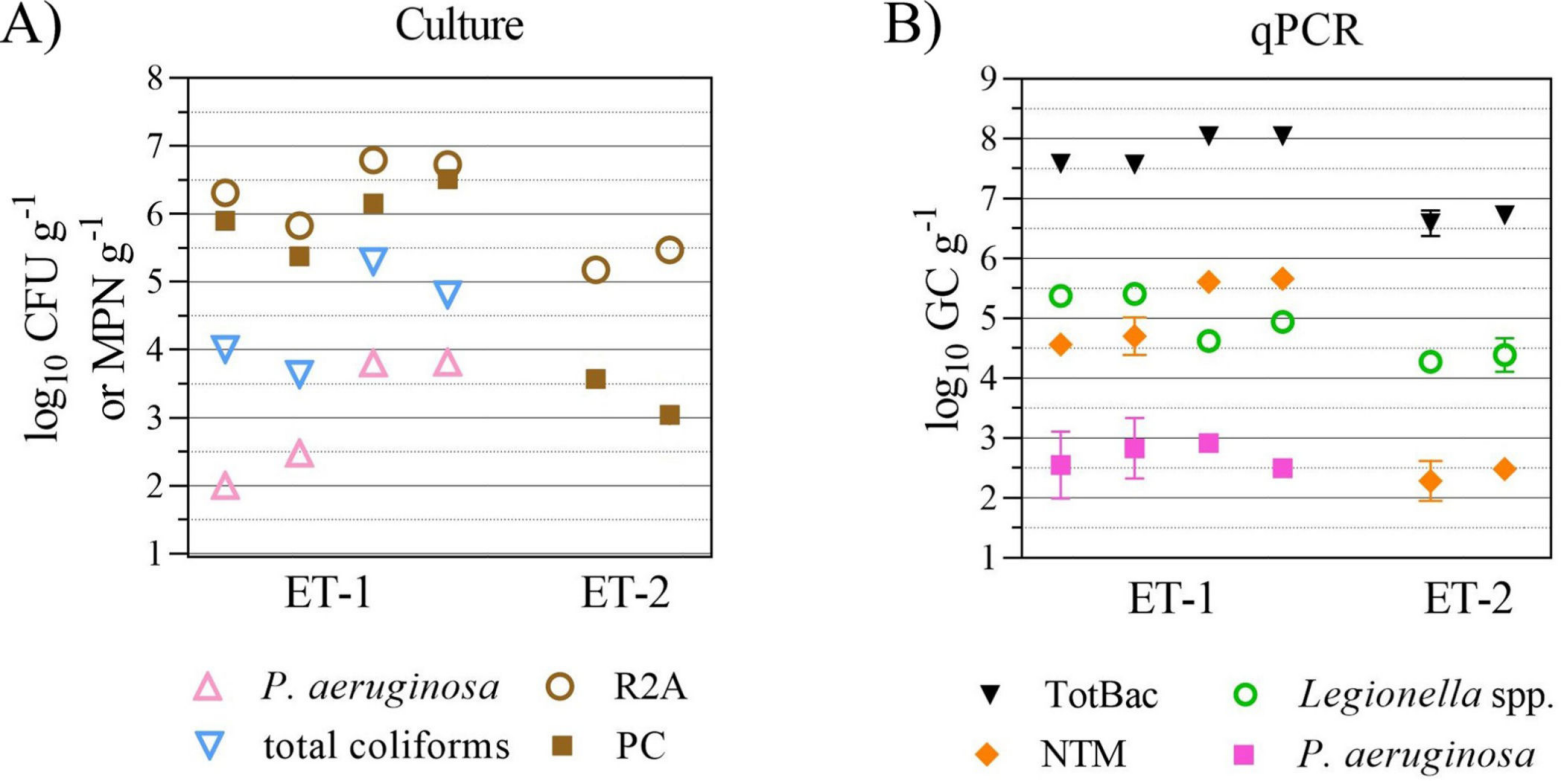
Culture (A) and qPCR (B) analysis of sediment samples from storage tanks ET-1 and ET-2. Culture results are expressed as mean log_10_ CFU g^−1^ for heterotrophic plate counts (using the R2A and PC methods) or MPN g^−1^ for *P*. *aeruginosa* and total coliform (TC). qPCR results are expressed as mean log_10_ gene copies (GC) g^−1^.

In preparation for the FCC, two elevated storage tanks, ET-1 and ET-1 (Fig 1A) were emptied and sediment samples from the bottom of the tank were collected and processed for culture and molecular analysis (Fig 3).

No culturable *Legionella* spp. was detected in these samples using the ISO 11731:2017 method. Using Legiolert, ET-2 samples were found to be negative for *L*. *pneumophila*; however, each of the four ET-1 samples displayed presumptive *L*. *pneumophila* positivity (turbidity and brown color). From those Legiolert trays, presumptive positive wells (25% of the large wells and 10% of the small wells) were extracted for confirmation tests. The wells were negative for *Legionella* spp. and *L*. *pneumophila* via PCR and after plating for bacterial isolates, colonies exhibited non-*Legionella*-like morphology on BCYE agar plates. Thus, the Legiolert presumptive positive wells were determined to be *L*. *pneumophila* false positives.

Culturable levels of R2A and PC HPC, total coliforms, and *P*. *aeruginosa* measured from the sediment samples are shown in Fig 3A. Average ± SD log_10_ CFU g^−1^ of PC and R2A HPC levels in ET-1 samples were 5.9±0.5 and 6.4±0.4; and in ET-2 samples, 3.3±0.6 and 5.3±0.2, respectively (Fig 3A, brown squares and circles). Unlike R2A HPC levels, PC HPC levels between ET-1 and ET-2 were statistically different (*P* < 0.0001) and most likely due to the ability of *P*. *aeruginosa* to be detected using the PC HPC method. *P*. *aeruginosa* and total coliforms were only detected in ET-1 samples at 3.0±0.9 and 4.4±0.8 log_10_ MPN g^−1^, respectively (Fig 3A, pink and blue triangles).

Sediment samples were all qPCR negative for *L*. *pneumophila* and three different free-living amoeba populations: *Acanthamoeba* spp., *Vermamoeba vermiformis*, and *Naegleria fowleri*. However, total bacteria, *Legionella* spp., nontuberculous mycobacteria (NTM), and *P*. *aeruginosa* were all detectable via qPCR in ET-1 and ET-2 sediment samples, except for *P*. *aeruginosa*, which was only detected in ET-1 (Fig 3B). In ET-1 sediment samples, average ± SD log_10_ gene copies (GC) g^−1^ levels of total bacteria were 7.6±0.3, 4.8±0.3 for *Legionella* spp., 4.9±0.5 for NTM, and 2.6±0.3 for *P*. *aeruginosa*. Statistically lower levels of total bacteria (6.4±0.02 log_10_ GC g^−1^, *P* = 0.003), *Legionella* spp. (4.1±0.2 log_10_ GC g^−1^, *P* = 0.03); and NTM (2.2±0.3 log_10_ GC g^−1^, *P* < 0.0001) were detected in ET-2 sediment samples, while no *P*. *aeruginosa* was detected in ET-2 sediments via qPCR (Fig 3B).

### 3.3 Microbiological quality of drinking water samples

#### 3.3.1 Bacterial enumeration and isolation.

**Fig 4.**
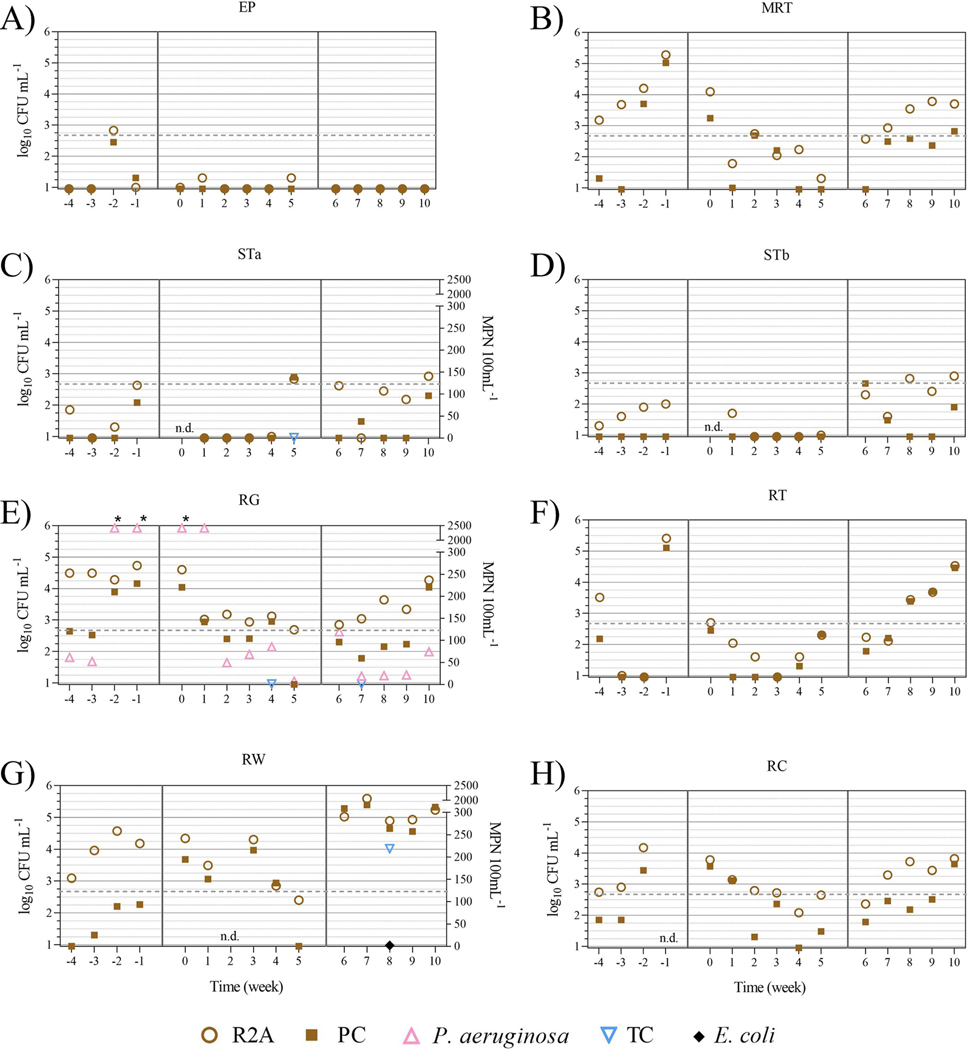
Culture analysis of water samples collected from distribution (A-D) and residential (E-H) sites. Heterotrophic plate counts (using the R2A and PC methods) are plotted on the left axis and expressed as log_10_ CFU mL^−1^. For the *P*. *aeruginosa*, total coliform (TC), and *E*. *coli* positive sites, STa (C), RG (E), and RW (G), culture results for are plotted on the right axis and expressed as MPN 100 mL^−1^. The grey dotted line indicates 500 CFU mL^−1^ or 2.7 log_10_ CFU mL^−1^. n. d., no data. *, levels were greater than the *P*. *aeruginosa* assay limit of 2,419.6 MPN 100mL^−1^.

Bulk water samples were analyzed for culturable HPCs, *Legionella* spp., *Pseudomonas aeruginosa*, total coliforms, and *Escherichia coli* (Fig 4). Although paired t-tests indicated HPC levels were statistically different between the R2A and PC methods, except for site EP (S1 Table), similar trends were observed for both at each location across the three sampling periods (Fig 4, brown circles and brown squares, respectively).

For all locations, HPC levels were generally lower during the FCC compared to the periods before and after. Average±SD log_10_ CFU mL^−1^ R2A HPC levels for distribution system and residential locations were 2.2±1.3 and 3.6±1.3 before, 1.5±0.8 and 2.8±0.9 during, and 2.2±1.0 and 3.8±1.0 after the FCC, respectively (Fig 4, brown circles). Similarly, average±SD log_10_ CFU mL^−1^ PC HPC levels for distribution system and residential locations were 1.6±1.2 and 2.4±1.2 before, 1.3±0.7 and 2.3±1.1 during, and 1.5±0.7 and 3.3±1.3 after the FCC, respectively (Fig 4, brown squares). There were no statistical differences in average HPC levels before, during, and after the FCC for each sampling location (*P* > 0.2), except for PC HPC levels at the RW location before and after the FCC (Fig 4G, 1.7±0.6 and 5.0±0.4 log_10_ CFU mL^−1^, respectively, *P* < 0.001).

No culturable *Legionella* spp. was detected throughout the study period using the ISO 11731:2017 method. Additionally, a subset of residential bulk water samples, at wk4 during the FCC, also tested negative for *L*. *pneumophila* using Legiolert. *P*. *aeruginosa* was only detected in bulk water samples from the RG location (Fig 4E, pink triangles). Levels ranged from 7.4 to >2,419.6 MPN 100mL^−1^ with the four highest levels of *P*. *aeruginosa* observed at wk-2 through wk1. Excluding those four highest *P*. *aeruginosa* levels, average ± SD MPN 100mL^−1^ levels before, during, and after the FCC were 57±7, 53±34, and 51±45, respectively. Total coliforms were detected in four bulk water samples during this study: wk5 at STa (1 MPN 100mL^−1^), wk4 and wk7 at RG (both 1 MPN 100mL^−1^), and wk8 at RW (219 MPN 100mL^−1^) with the latter sample also being *E*. *coli* positive at a concentration of 2 MPN 100mL^−1^ (Fig 4C, 4E, and 4G: blue triangles and black diamond).

#### 3.3.2 Bacterial isolate analysis via whole genome sequencing.

Presumptive *P*. *aeruginosa*, total coliform, and *E*. *coli* culture isolates were processed for whole-genome sequence analysis to identity and confirm their genetic lineage and to better understand their molecular diversity and occurrence within the distribution system. Sequenced isolates included a subset of seven *P*. *aeruginosa* strains, six cultured from the RG water samples and one from ET-1 sediments; six total coliform strains; and one *E*. *coli* strain. All were taxonomically identified as shown in Table 1.

**Fig 5.**
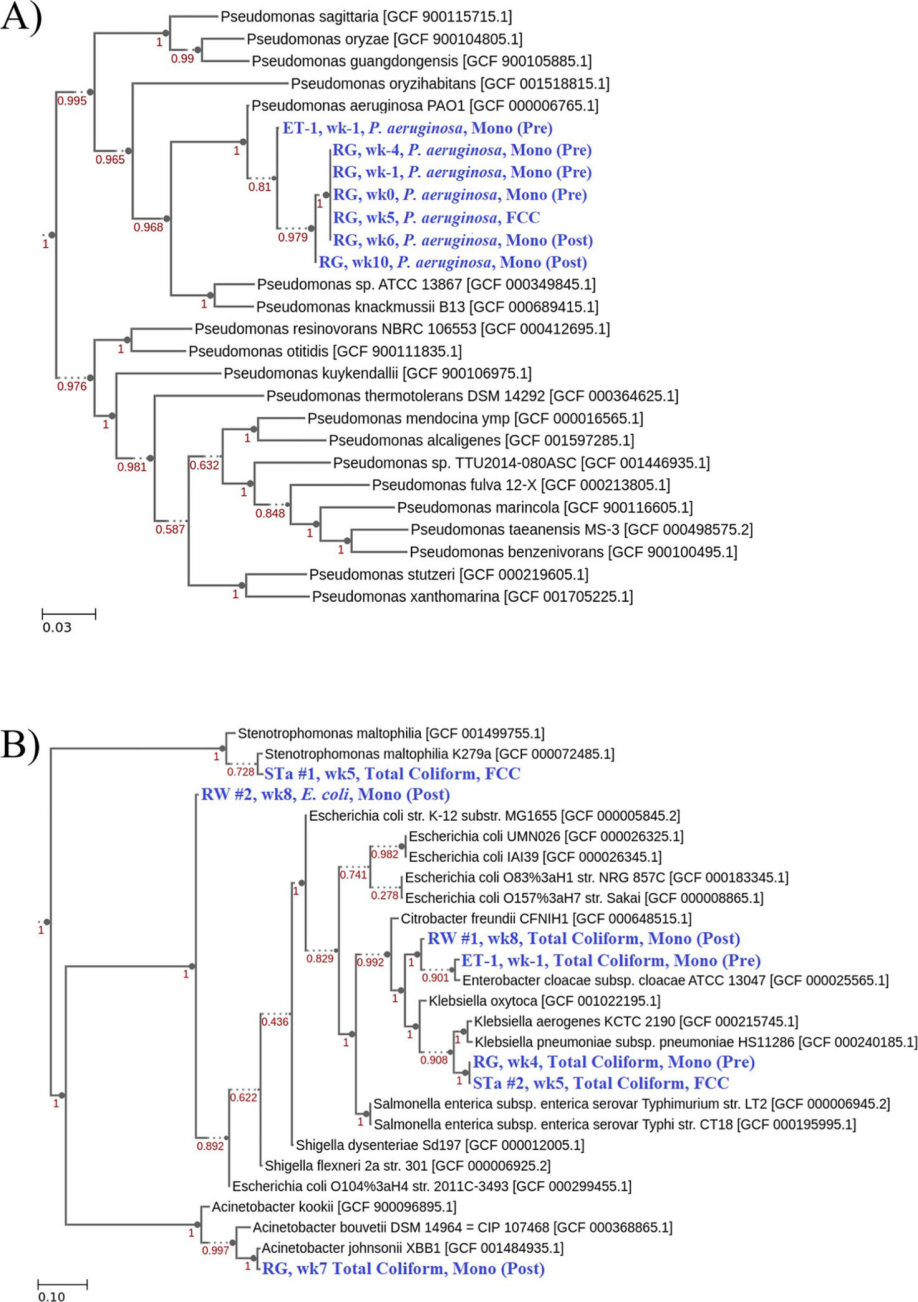
Phylogenetic trees illustrating isolate relatedness to reference genomes. Representative *P*. *aeruginosa* (A), total coliform and *E*. *coli* (B) isolates from each sampling location and time were chosen for construction of these phylogenetic trees. Isolate genomes (blue-colored text) are described by the sampling location, sampling week, presumptive isolate type, and disinfectant residual period during isolation. Branch support values (red-colored text) are shown for each node and represent confidence values (bootstrapping) used by FastTree2 to estimate maximum likelihood. The scale bar represents the phylogenetic distance of 0.03 (A) or 0.10 (B) nucleotide substitutions per site.

The seven *P*. *aeruginosa* isolates were confirmed as *P*. *aeruginosa* with the % average nucleotide identity (ANI) to reference *P*. *aeruginosa* genomes ranging from 99.4 to 99.5% (Table 1). A phylogenetic tree was constructed and showed that the RG Pa isolates were all located on the same branch except for the one isolated during the last week of the study and a month after the switch back to chloramine disinfection (RG, wk10, Mono(Post), Fig 5A).

The ET-1 storage tank sediment sample, collected one week prior to the free chlorine conversion, was found to be total coliform positive (Fig 3A, blue triangle). This ET-1 total coliform isolate, wk-1 Mono (Pre), was identified as *Enterobacter ludwigii* with an average nucleotide identity (ANI) of 99.0% to reference genomes (Table 1). Another *Enterobacter* total coliform species, *Enterobacter hormaechei*, was isolated nine weeks later from the RW residential bulk water sample (Fig 4G, blue triangle; Table 1, RW #1, 99.0% ANI). RW #1 was isolated at wk8 Mono (Post), which was the period after the FCC and two weeks after the return to chloramine disinfection (Fig 1). Phylogenetic analysis indicated relatedness between the ET-1 *E*. *ludwigii* and the RW *E*. *hormaechei* isolates (Fig 5B and S5A Fig). The RW wk8 sample was also *E*. *coli* positive (Fig 4G, black diamond) and genetically confirmed with an ANI of 97.0% (Table 1, RW #2; S5B Fig).

The wk5 STa water sample, collected during the free chlorine conversion, was also positive for total coliforms (Fig 4C, blue triangle). However, during the isolation for pure bacterial colonies, two different colony morphologies were observed on the TSA plates with STa colony type #1, identified as *Stenotrophomonas maltophilia* with 97.8% ANI, and STa colony type #2, as *Raoultella planticola* with 99.3% ANI (Table 1, S5C and S5D Fig). Notably, *R*. *planticola* was also isolated from the RG water sample, collected during the free chlorine conversion at wk4 (Fig 3E, blue triangle; Table 1, 99.3% ANI). Phylogenetic analyses indicated that both wk4 RG and wk5 STa *R*. *planticola* isolates were closely related (Fig 5B and S5D Fig). Another RG water sample was also total coliform positive, at wk7 collected two weeks after the water system switched back to applying a chloramine residual (Fig 4E) but was identified as *Acinetobacter johnsonii* with 95.9% ANI (Table 1; Fig 5B and S5E Fig).

## 4. Discussion

In chloraminated drinking water systems (CDWSs), activity of nitrifying bacteria and their microbial products results in both unstable, and loss of, chloramine residuals throughout portions of the distribution system [36, 37]. For the surface water CDWS evaluated in this study, the State Primacy Agency requires water systems to implement a nitrification control plan that describes target levels along with monitoring and follow up actions to control nitrification. When the onset of nitrification occurs, a FCC is recommended for remediation. Moreover, CDWSs in this State typically implement a FCC either following the detection or suspected occurrence of *Naegleria fowleri* in the system; addressing nitrification events (e.g., elevated ammonia, nitrite, and/or nitrate levels; and/or when disinfection residuals are not within the minimum requirements for CDWSs in the State (e.g., 0.5ppm).

During the start of this study, the CDWS suspected *N*. *fowleri* occurrence in their system due to the heavy and sustained rainfall events in the Spring of 2021. Previous studies have demonstrated the occurrence of *N*. *fowleri* in roof harvested rainwater and that surface waters can become contaminated with *N*. *fowleri* due to soil runoff after rainfall events [38, 39]. Moreover, recreational usages of treated water (e.g., residential lawn water slide, water park/splash pad) have been previously reported as sources of *N*. *fowleri* infections and deaths in young children [40, 41]. Thus, the CDWS planned for a 60-day FCC and water samples were collected from the distribution system and sent to a commercial *N*. *fowleri* analytical lab by the State during the first week of the FCC. Additionally, 1 L of water samples collected at wk1 from all 8 study locations (Fig 1A) were concentrated and analyzed by the USEPA for *N*. *fowleri* using qPCR targeting the 18S rRNA gene [25]. Both State and USEPA samples were culture- and qPCR-negative for *N*. *fowleri*; thus, the CDWS reduced duration of the FCC to 42 days.

Previous studies have reported nitrification reoccurring within 10 to 16 weeks after systems have implemented a 4 to 6 weeklong FCC suggesting benefits of this practice may be temporary [42–44]. Absence of chloramine residual and increased nitrate in the system was observed as early as 12 weeks after the FCC [42]. Ammonia-oxidizing bacteria (AOB) were detected within pipe wall biofilms during, and at the end of, the FCC [42]; bacterial cell counts increased within days after the FCC [45]; continuous rapid regrowth of nitrifying bacteria was observed 16 weeks after the FCC [43]; and bacterial cell counts and nitrite levels increased significantly 10 weeks after the FCC [44]. Collectively, these studies demonstrate that FCCs may not completely and effectively remove nitrifying bacteria and may not confer long-term nitrification control after the return to chloramine secondary disinfection. Thus, CDWSs may need to customize and/or manage their implementation of a FCCs differently to maximize its benefits for their system.

The heterotrophic plate count (HPC) method is used for enumeration of culturable heterotrophic bacteria in water and serves as a general assessment of drinking water quality [46]. Reasoner’s 2A (R2A) and Plate Count (PC) agar methods were evaluated in this study to quantify slow-growing, water-based bacteria versus fast growing, more fastidious, higher temperature tolerant heterotrophic bacteria, respectively [19, 46]. Although R2A HPCs were statistically higher than PC levels, similar trends were observed for both at each location across the three sampling periods (Fig 4). Average HPC levels at the MRT and residential sites, gradually decreased from the first to sixth week during the FCC (4.2 to 2.3 log_10_ CFU mL^−1^, respectively), but then quickly increased to similar or higher levels from the first to fifth week after the FCC (3.2 to 4.3 log_10_ CFU mL^−1^, respectively) indicating that free chlorine was able to better control and limit HPC growth compared to chloramine. Moreover, the return to high HPC levels shortly after the FCC observed in this study, may also suggest the short-term benefits of the FCC (Fig 4). However, other factors can contribute to high HPC levels (e.g., temperature, availability of oxygen and nutrients, pH, etc.); thus, increases in HPC levels alone are not indicative of nitrification [15].

Opportunistic pathogens such as *Legionella pneumophila* and *Pseudomonas aeruginosa* are significant public health concerns especially given their frequent occurrence in, and persistent colonization of, premise plumbing systems [47, 48]. Thus, both pathogens were monitored during this study to evaluate their occurrence in both the distribution and premise plumbing residential sites. While no culturable *Legionella* spp. was detected for the entire study period, all RG samples were culture positive for *P*. *aeruginosa* (Fig 4E). The four highest levels of culturable *P*. *aeruginosa*, (≥ 2,419 MPN 100mL^−1^) were observed during each of the two weeks before and two weeks after initiation of the FCC. The lowest level (7 MPN 100mL^−1^) occurred during the last week of the FCC and the second highest (120 MPN 100 mL^−1^) occurred two days after the switch back from free chlorine to chloramine (Fig 4E).

These observations are supported by Xue *et al*. [49] which reported the high reactivity of *P*. *aeruginosa* extracellular polymeric substances (EPS) to chlorine compared to low reactivity with monochloramine. The mechanism was attributed to EPS shielding of the *P*. *aeruginosa* cell surface preventing monochloramine access to disinfection reactive sites on the bacterial cell membrane [49]. Notably, the plumbing under the RG kitchen sink is comprised of flexible plastic and copper pipe material while RT is comprised of braided polymer, plastic and copper piping, RW shows a mixture of copper, plastic, and braided polymer piping, and RC plumbing consists of copper, braided polymer, and cross-linked polyethylene (PEX) materials (S1 Fig). Monochloramine disinfection of biofilms has been shown to be less effective on PVC surfaces compared to copper, and conversely, free chlorine disinfection was shown to be more effective on PVC compared to copper biofilms [10]. However, it is unclear how plumbing configurations composed of mixed plastic and metal piping materials would impact disinfection efficacy. Although no single chemical and physiochemical water quality parameter could explain the levels of *P*. *aeruginosa* observed at each corresponding time point, it is likely a combination of environmental factors, water quality parameters, and bacterial traits contributed to the growth patterns of *P*. *aeruginosa* observed in the RG samples.

To genetically characterize the *P*. *aeruginosa* detected in the RG and ET-1 samples, total genomic DNA from six RG *P*. *aeruginosa* isolates obtained before, during, and after the FCC, and one isolate from ET-1 was processed for whole-genome sequencing. The *P*. *aeruginosa* genome sizes were between 6.3 to 7 million base pairs (Mbp) (Table 1) which is within the range of 5.5 to 7 Mbp observed in previously sequenced isolates [50]. The RG strains were genetically similar except for the RG wk10 isolate obtained 5 weeks after the FCC, as indicated by the separation onto another branch in the phylogenetic tree (Fig 5A). Based on RAST and SEED analyses, these genetic differences between RG wk10 and the other RG isolates included a higher number of protein domains associated with cell wall synthesis and DNA repair; and less domains associated with motility and phenazine biosynthesis, which has been shown to play an important role in gene expression and antibiotic tolerance [51].

For the ET-1 *P*. *aeruginosa* isolate, separation from the RG isolates was due to the higher amount of protein coding domains associated with amino acid, carbohydrate, DNA, protein, and RNA metabolism; heat shock and oxidative stress responses; iron acquisition and metabolism; membrane transport; cellular respiration; and denitrification. Under anaerobic conditions and in the presence of nitrate, *P*. *aeruginosa* can perform complete denitrification by reducing nitrate to molecular nitrogen via nitrite, nitric oxide, and nitrous oxide utilizing the enzymes: nitrate reductase, nitrite reductase, nitric oxide reductase, and nitrous oxide reductase [52]. The nitrogen oxides are utilized by *P*. *aeruginosa* as alternative electron acceptors that enable their growth under anaerobic conditions [53]. The ET-1 *P*. *aeruginosa* isolate, compared to the residential isolates, contained higher numbers of protein domains associated with nitrite reductase as well as two enzymes, formate dehydrogenase and nicotinamide adenine dinucleotide hydrogen (NADH)-quinone oxidoreductase, which are involved with cellular respiration [53]. Utilizing drinking water system sediment deposits, Liu *et al*. [54] showed that nitrification occurs within the oxic, or oxygen containing, zone; while denitrification occurs in the anoxic, or oxygen depleted, zone. Thus, the genetic traits unique to ET-1 *P*. *aeruginosa* most likely plays an important role in supporting and enabling their growth in storage tank sediment environment.

Molecular detection of several bacterial and eukaryotic opportunistic pathogens was also performed on the ET-1 and ET-2 sediment samples. *L*. *pneumophila* and the free-living amoebae, *Acanthamoeba* spp., *Vermamoeba vermiformis*, and *Naegleria fowleri* were not detected in these samples by qPCR. However, total bacteria, *Legionella* spp, and nontuberculous mycobacteria (NTM), a group that includes opportunistic pathogens, were detected in ET-1 and ET-2; while *P*. *aeruginosa* was only detected in ET-1 (Fig 3B). Combining ET-1 and ET-2 levels, average±SD log_10_ GC g^−1^ levels of total bacteria were 7.2±0.6 log_10_ GC g^−1^; for *Legionella* spp., 4.6 ±0.5 log_10_ GC g^−1^; for NTM, 4.0±1.5 log_10_ GC g^−1^; and for *P*. *aeruginosa*, 2.6±0.3 log_10_ GC g^−1^. Except for *P*. *aeruginosa*, the opportunistic pathogen levels observed in this study were lower than those reported previously during a large survey of 87 sediment samples collected from 18 locations across 10 US states [55], which was most likely due to differences in sample number, sediment collection and processing methodology, and qPCR assay targets between studies. However, the reproducible detection of opportunistic pathogens in storage tank sediment samples further highlights their role as potential reservoirs of human pathogens. Thus, these results advocate for better operations and management of water storage structures (e.g., more frequent inspections, regular cleaning/removal of debris, biofilm build-up, and sediment, along with addressing identified sanitary defects such as missing/damaged screens and gaskets, unprotected holes, and assessment of the structural integrity) to reduce the risk of widespread contamination in distributed bulk water and to ensure public health protection from these appurtenances.

Total coliforms were also isolated from the ET-1 sediment samples as well as from RG at wk4 and STa at wk5, during the FCC; and from RG at wk7 and RW at wk8, which was also *E*. *coli* positive, after the FCC (Fig 4). Table 1 shows the taxonomic identification of the six total coliform isolates which were identified as *Stenotrophomonas maltophilia* (from STa), *Raoultella planticola* (from STa and RG), two members of the *Enterobacter cloacae* complex, *E*. *ludwigii* and *E*. *hormaechei* (from ET-1 and RW, respectively), and *Acinetobacter johnsonii* (from RG), all of which have been associated with human infections and are considered opportunistic pathogens [56–60]. Due to the sporadic detection of total coliforms and *E*. *coli* at the various locations during this study, the positives detected may have been a result of their temporal and spatial proximity to boil advisory issuing events and not related to the FCC.

The time span, proximity, and reason for the boil advisories related to the TC and *E*. *coli* positive locations were: for the RG wk4 detect, a main repair that took place 2.3 mi south of the RG site 10 days prior to sampling; for the STa wk5 detect, a location experienced a pressure loss requiring a hydrant repair 10 days prior to sampling and 7.5 mi north of STa site; for the RG wk7 detect, an equipment failure occurred at a pumping station 11.2 mi southwest of the RG location four days prior to sampling; and for the RW wk8 detect, a transmission line was repaired due to pressure loss at a location 10 days prior to sampling and 6 mi north of the RW site. However, correlations between total coliform and *E*. *coli* detection and water quality changes because of the FCC cannot be ruled out, especially since total coliforms and *E*. *coli*were not detected before the FCC.

Statistical relationships between levels of total coliforms and disinfectant residual in treated drinking water is a current knowledge gap that needs to be addressed to ensure microbial regrowth is controlled and microbial water quality is maintained throughout the water distribution network. Sporadic detection of total coliforms adds to the challenge of establishing those statistical correlations on top of the delay due to incubation times needed for total coliform culture analyses. Rapid detection of coliform bacteria and the ability to quickly identify potential contamination sources will help maximize public health protection from microbial regrowth and contamination within the distribution system. Thus, future studies should explore monitoring total coliforms throughout distribution and premise plumbing systems using molecular techniques and evaluate their use as a rapid and specific test for coliform bacteria while monitoring for disinfectant residuals. Several PCR assays have been developed for total coliform detection and tested with over 150 total coliform strains representing 76 species using potable ground and surface water samples and confirmed with standard TC culture methods [61–64].

In addition to the chemical and microbial parameters collected during this study, future FCC studies should also incorporate molecular monitoring of ammonia oxidizing bacteria (AOB), ammonia oxidizing archaea (AOA), nitrite oxidizing bacteria (NOB), and complete ammonia oxidation (commamox) groups and species to identify significant changes in, and responses of, nitrifying populations during a FCC. The differences in physio- and chemical water quality and genetic diversity of culturable opportunistic and nosocomial pathogens, observed in this study, highlights the complexity of water quality changes that can occur during implementation of FCCs. Accordingly, FCC practices should be evaluated to ensure regulatory compliance while maximizing long-term water quality benefits and public health protection.

## 5. Summary

- Based on the microbial culture results, the beneficial impacts of the FCC conducted in this study may have been temporary.
- Monitoring for genetic diversity of pathogens throughout a distribution system can provide mechanistic insights into their occurrence and reveal specific niches/habitats and their genetic adaptations to those environments such as with the ET-1 *P*. *aeruginosa* isolate analyzed in this study. The conducive growth environments could then be removed or managed with targeted treatment or operational practices to control and prevent their growth in those environments.
- Utilizing molecular methods for total coliform detection could reveal correlative links and insights between their occurrence and disinfectant residual concentrations.
- Due to the frequent use of FCCs, similar monitoring studies should be conducted to aid water treatment operators and managers in optimizing their standard practice or perhaps replace or supplement with other practices (e.g., flushing) that can maximize benefits of the FCC.

## Supplementary Material

Supplementary Material **SI Fig. Kitchen faucet type, plumbing materials, and configuration for residential sites.** Images were taken by homeowners for the RG (A), RT (B), RW (C), and RC (D) kitchen sampling location used in this study. The under sink plumbing consists of: plastic hoses from the valve to the fixture with the valve connected to copper plumbing at RG (A); braided polymer tubing from the valve to fixture with the valve is connected to copper plumbing at RT (B); braided polymer and plastic tubing from the valve to fixture with the valve connected to copper plumbing at RW (C); and cross-linked polyethylene (PEX) and copper materials from the valve to fixture with the valve is connected to copper plumbing at RC (D). Note that drain line materials and components are not included in this plumbing materials list. Published with permission from the USEPA Region 6 participating drinking water utility.
**S2 Fig. Processing of storage tank sediment samples.** Sediment samples from ET-1 were collected into four bottles (A) and from ET-2 into two bottles (B). The liquid phase from each bottle (C, D) was decanted into separate sterile containers (e.g., glass 1L bottle shown on the left side of panels C and E). Small aliquots of the liquid phase were placed in 50mL conical tubes. Settled and resuspended particles in the sediment liquid phase are shown for ET-1 (E) and ET-2 (F).
**S3 Fig.** Temporal physiochemical parameters summary for distribution (A-C) and residential (D-F) sites. Sampling occurred weekly before (wk-4 to −1), during (wk0 to 5), and after (wk6 to 10) the FCC period. pH (A, E), temperature (B, F), turbidity (C, G), and hardness (D, H) levels are shown in the down triangle, hexagon, star symbols, and cross-hatched circle symbols, respectively. Each sampling location is represented by different colors (EP, black; MRT, grey; STa, light green; STb, light blue; RG, pink; RT, orange; RW, dark green; RC, dark blue). nd, no data, for STa and STb during week 5, for RC during week 4, and for RW during week 7.
**S4 Fig.** Temporal free ammonia, nitrite, and orthophosphate level summary for distribution (A-C) and residential (D-F) sites. Sampling occurred weekly before (wk-4 to −1), during (wk0 to 5), and after (wk6 to 10) the FCC period. Free ammonia (A, D), nitrite (B, E), and orthophosphate (C, F) levels are shown in the diamond, cross-hatched squares, and X, respectively. Each sampling location is represented by different colors (EP, black; MRT, grey; STa, light green; STb, light blue; RG, pink; RT, orange; RW, dark green; RC, dark blue). nd, no data, for STa and STb during week 5, for RC during week 4, and for RW during week 7.
**S5 Fig. Phylogenetic trees based on total coliform classifications.** Phylogenetic trees based on *Enterobacter* (A), *E*. *coli* (B), *Stenotrophomonas* (C), *Raoultella* (D), and *Acinetobacter* (E) classifications of study isolates are shown. Genomes (blue-colored text) are described by the sampling location, sampling week, presumptive isolate type, and disinfectant residual period during isolation. Branch support values (red-colored text) are shown for each node and represent confidence values (bootstrapping) used by FastTree2 to estimate maximum likelihood. The scale bar represents the phylogenetic distance of either 0.01 (A, B, D), 0.02 (E), or 0.04 (C) nucleotide substitutions per site.
S1 Table. Two-tailed paired t-test summary for culturable HPC levels using the R2A and PC methods.

## Figures and Tables

**Table 1.** Summary statistics of whole-genome assemblies for the drinking water and storage tank isolates.

Isolate Origin			Taxonomic Identification		Genome size (bp)	No. of contigs	Contig *N*_50_ (bp)	G+C content (%)	No. predicted genes	No. protein coding genes	NCBI BioSample Accession No.^a^
Sampling location	Time point	Period	Predicted organism	ANI (%)							
ET-1	wk-1	Mono (Pre)	*Pseudomonas aeruginosa*	99.4	6,321,307	266	48,402	66	5,837	5,767	SAMN29983815
RG	wk-4	Mono (Pre)	*Pseudomonas aeruginosa*	99.5	6,994,078	112	221,566	66	6,496	6,423	SAMN29983816
RG	wk-1	Mono (Pre)	*Pseudomonas aeruginosa*	99.5	6,993,886	111	221,566	66	6,492	6,423	SAMN29983817
RG	wk0	Mono (Pre)	*Pseudomonas aeruginosa*	99.5	6,994,629	109	222,231	66	6,483	6,413	SAMN29983818
RG	wk5	FCC	*Pseudomonas aeruginosa*	99.5	6,994,701	107	259,311	66	6,489	6,416	SAMN29983819
RG	wk6	Mono (Post)	*Pseudomonas aeruginosa*	99.5	6,994,677	110	183,537	66	6,500	6,430	SAMN29983820
RG	wk10	Mono (Post)	*Pseudomonas aeruginosa*	99.5	6,995,519	264	58,435	66	6,493	6,422	SAMN29983821
ET-1	wk-1	Mono (Pre)	*Enterobacter ludwigii*	99.0	4,936,203	120	81,037	54	4,627	4,558	SAMN29983828
STa #1	wk5	FCC	*Stenotrophomonas maltophilia*	97.8	4,762,406	64	145,667	66	4,430	4,357	SAMN29983823
STa #2	wk5	FCC	*Raoultella planticola*	99.3	5,483,668	32	492,012	56	5,131	5,062	SAMN29983824
RG	wk4	FCC	*Raoultella planticola*	99.3	5,483,859	30	497,847	56	5,128	5,060	SAMN29983822
RG	wk7	Mono (Post)	*Acinetobacter johnsonii* ^a^	95.9	3,419,695	70	84,111	41	3,357	3,305	SAMN29983825
RW #1	wk8	Mono (Post)	*Enterobacter hormaechei*	99.0	4,654,922	113	79,059	56	4,399	4,325	SAMN29983826
RW #2	wk8	Mono (Post)	*Escherichia coli*	97.0	4,956,538	1,323	97,970	48	7,198	7,099	SAMN29983827

Abbreviations: ANI, average nucleotide identify; bp, base pair; FCC, free chlorine conversion; G+C, guanine-cytosine content; Mono (Pre), monochloramine residual period prior to FCC; Mono (Post), monochloramine residual period after the FCC; NCBI, National Center for Biotechnology Information; No., number.

a Sequences are deposited under NCBI BioProject PRJNA871216.

## Data Availability

Raw data is publicly deposited on the USEPA’s ScienceHub website (https://catalog.data.gov/harvest/epa-sciencehub). The Illumina raw sequence reads are deposited in the National Center for Biotechnology Information (NCBI) Sequence Read Archive (SRA) database under the BioProject accession number PRJNA871216 (Temporary Submission ID: SUB11952375 Release date: 2023–10-31). https://www.ncbi.nlm.nih.gov/sra/.
