# Supplementary material for: Microbial and physicochemical water quality changes within distribution and premise plumbing systems during a chlorine conversion
Source: PLOS Water. Author manuscript; Available in PMC 2025 Apr 9. (PMC11980844; doi:10.1371/journal.pwat.0000181)
Supplement: Supplementary Material — SI Fig. Kitchen faucet type, plumbing materials, and configuration for residential sites. Images were taken by homeowners for the RG (A), RT (B), RW (C), and RC (D) kitchen sampling location used in this study. The under sink plumbing consists of: plastic hoses from the valve to the fixture with the valve connected to copper plumbing at RG (A); braided polymer tubing from the valve to fixture with the valve is connected to copper plumbing at RT (B); braided polymer and plastic tubing from the valve to fixture with the valve connected to copper plumbing at RW (C); and cross-linked polyethylene (PEX) and copper materials from the valve to fixture with the valve is connected to copper plumbing at RC (D). Note that drain line materials and components are not included in this plumbing materials list. Published with permission from the USEPA Region 6 participating drinking water utility. S2 Fig. Processing of storage tank sediment samples. Sediment samples from ET-1 were collected into four bottles (A) and from ET-2 into two bottles (B). The liquid phase from each bottle (C, D) was decanted into separate sterile containers (e.g., glass 1L bottle shown on the left side of panels C and E). Small aliquots of the liquid phase were placed in 50mL conical tubes. Settled and resuspended particles in the sediment liquid phase are shown for ET-1 (E) and ET-2 (F). S3 Fig. Temporal physiochemical parameters summary for distribution (A-C) and residential (D-F) sites. Sampling occurred weekly before (wk-4 to −1), during (wk0 to 5), and after (wk6 to 10) the FCC period. pH (A, E), temperature (B, F), turbidity (C, G), and hardness (D, H) levels are shown in the down triangle, hexagon, star symbols, and cross-hatched circle symbols, respectively. Each sampling location is represented by different colors (EP, black; MRT, grey; STa, light green; STb, light blue; RG, pink; RT, orange; RW, dark green; RC, dark blue). nd, no data, for STa and STb during week 5, for RC during week 4, and for [file NIHMS2054355-supplement-Supplementary_Material.zip › pwat.0000181.s006.pdf]

S1 Table. Two-tailed paired t-test summary for culturable HPC levels using the R2A and PC methods

Paired t test: R2A v PC HPC levels	Location							
	**EP**	**MRT**	**STa**	**STb**	**RG**	**R**	**RW**	**RC**
***P* value**	0.2447	0.0004	0.0243	0.0062	<0.0001	0.0178	0.0062	<0.0001
***P* value summary**	ns	***	*	**	****	*	**	****
**Significantly different (*P* < 0.05)?**	No	Yes	Yes	Yes	Yes	Yes	Yes	Yes
**One- or two-tailed *P* value?**	Two-tailed	Two-tailed	Two-tailed	Two-tailed	Two-tailed	Two-tailed	Two-tailed	Two-tailed
**t, df**	t=1.214, df=14	t=4.682, df=14	t=2.548, df=13	t=3.259, df=13	t=5.381, df=14	t=2.684, df=14	t=3.264, df=13	t=6.260, df=13
**Number of pairs**	15	15	14	14	15	15	14	14
**Mean of differences**	-0.05533	-0.9233	-0.4486	-0.5636	-0.8853	-0.2953	-0.8807	-0.7943
**SD of differences**	0.1765	0.7638	0.6587	0.6469	0.6372	0.4261	1.01	0.4747
**SEM of differences**	0.04556	0.1972	0.1761	0.1729	0.1645	0.11	0.2698	0.1269
**95% CI**	-0.153 to 0.042	-1.346 to -0.500	-0.829 to -0.068	-0.937 to -0.190	-1.238 to -0.532	-0.531 to -0.059	-1.464 to -0.298	-1.068 to -0.520
**R squared (partial eta squared)**	0.0953	0.6102	0.3331	0.4497	0.6741	0.3398	0.4504	0.7509

Abbreviations: CI, confidence interval; df, degrees of freedom; R, correlation coefficient; SD, standard deviation; SEM, standard error mean
